# Synergistic Effect of Enzyme‐Assisted Fermentation on Phytochemical Content, Volatile Profile, and Antioxidant, Antidiabetic, and ACE Inhibitory Properties of Sea Buckthorn‐Based Cereal Beverages

**DOI:** 10.1002/fsn3.71217

**Published:** 2025-11-28

**Authors:** Afusat Yinka Aregbe, Shamas Murtaza, Ebenezer Ola Falade, Md. Hafizur Rahman, Yongkun Ma

**Affiliations:** ^1^ School of Food and Biological Engineering Jiangsu University Zhenjiang China; ^2^ Department of Food Science and Technology MNS University of Agriculture Multan Multan Pakistan; ^3^ Zhejiang University‐Zhongyuan Institue Zhengzhou Henan China; ^4^ Department of Quality Control & Safety Management, Faculty of Food Sciences & Safety Khulna Agricultural University Khulna Bangladesh

**Keywords:** aroma profile, bioactivity, enzyme‐assisted fermentation, functional beverage, phytochemical content, sea buckthorn

## Abstract

Today, plant‐based beverages are increasingly being recognized for their health‐promoting properties. Here, we investigate how the innovative use of enzyme‐assisted fermentation enhances the phytochemical content, bioactivity, and aroma profile of sea buckthorn‐based cereal beverages (SBCB). Enzyme‐assisted fermentation significantly increased phytochemical content compared to the untreated sample. Specifically, F3% (fermented, 3% enzyme‐treated SBCB) boosted total phenolic and flavonol contents by 124.94% and 147.24%, while F5% (fermented, 5% enzyme‐treated SBCB) raised total flavonoid and proanthocyanidin contents by 177.74% and 58.32%. Notably, F5% showed the most pronounced improvements in antioxidant, antidiabetic, and ACE inhibitory effects. Analysis from the Mantle test showed that low pH and enzyme activity contributed to increased phytochemical content and enhanced bioactivity, while the aroma analysis revealed higher levels of alcohols and acids (e.g., 3‐methyl butan‐1‐ol, acetic acid) alongside reduced ester content in the enzyme‐treated fermented samples. Together, our findings demonstrate enzyme‐assisted fermentation as an innovative strategy for developing fruit‐based cereal beverages with superior functional properties.

## Graphical abstract

Enzyme‐assisted fermentation of sea buckthorn–based cereal beverages (SBCB) markedly elevated extractable phenolics and flavonoids (F3%: TPC +124.9%, TFLC +147.2%; F5%: TFC +177.7%, TPAC +58.3%) alongside stronger antioxidant, antidiabetic, and ACE inhibitory activities. Mantel analysis linked these gains to lower pH and enzyme activity; volatile profiling showed reduced esters and higher alcohols, revealing a distinct shift in the volatile profile. Enzyme‐assisted fermentation provides a robust, scalable strategy to produce SBCB with enhanced functional properties.

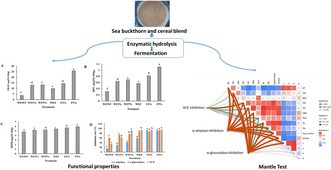

## 1 Introduction

As noncommunicable diseases (NCDs) such as diabetes, hypertension, and cancer become more prevalent globally, the importance of healthier dietary patterns, rich in whole foods and nutrients, is gaining attention. The therapeutic potential and bioactive compounds of plant‐based foods have made them a crucial part of a healthy diet (Fernandes et al. 2018; Tachie et al. 2023). This trend has spurred a growing demand for functional plant‐based beverages, driving the transformation of indigenous plant raw materials into innovative products that meet health and sustainability goals. Cereals, widely available and rich in macronutrients, represent a promising raw material for functional beverages (Fernandes et al. 2018). However, their inherent nutrient variability and limited bioavailability of bioactive compounds present challenges to achieving optimal product quality. By combining cereals with fruits rich in vitamins, minerals, and phytochemicals, plant‐based beverages can be made more nutritious, appealing, and consumer‐friendly.

Sea buckthorn ( *Hippophae rhamnoides* ), a fruit native to China, has been utilized for centuries due to its nutritional and medicinal benefits. It is gaining global recognition for its abundant bioactive compounds and nutrients, including vitamins, essential fatty acids, carotenoids, and phenolic compounds, particularly flavonoids (El‐sohaimy et al. 2022; Dubey et al. 2023). Studies have shown that it possesses a range of biological activities, including anti‐inflammatory, antimicrobial, anticancer, and antioxidant effects, as well as reducing blood sugar, cholesterol, and blood pressure (El‐sohaimy et al. 2022; Dubey et al. 2023; Peng et al. 2023). However, the bioavailability and biological activity of many phytochemicals are limited because they are structurally bound to the plant cell wall. To overcome this limitation, advanced processing methods such as enzymatic hydrolysis and fermentation are essential to release these compounds and enhance their bioactivity.

For centuries, fermentation has been employed to improve the nutritional, organoleptic, and functional attributes of plant‐based foods and beverages (Liu et al. 2017; Lao et al. 2020; Guo 2024). This process involves the use of microorganisms to transform phytochemicals into more bioactive forms, thereby enhancing the health benefits of the final product (Liu et al. 2017). Enzymatic hydrolysis, a complementary process, effectively degrades plant cell walls, releasing phenolic compounds and improving their bioavailability (Liu et al. 2017). Although these individual processing methods have proven effective, combining them could yield better results (Liu et al. 2017; Guo 2024). The integration of enzymatic hydrolysis with fermentation introduces a novel, synergistic approach that maximizes the release and transformation of bioactive compounds, such as polyphenols and flavonoids, into more bioavailable forms. This dual‐process innovation not only enhances the functional properties of plant‐based beverages but also aligns with modern consumer demands for health‐oriented and sustainable products (Milić et al. 2023). Studies on rice bran (Liu et al. 2017) and spent coffee grounds (Milić et al. 2023) have demonstrated that combining enzymatic hydrolysis and fermentation significantly increases bioactive content and enhances the therapeutic potential and sensory properties of these plant‐based foods.

Research has examined the individual and combined effects of fermentation and enzymatic hydrolysis on cereals and sea buckthorn (Liu et al. 2017; Lao et al. 2020; Guo 2024), but the synergistic impact of these processes on fruit‐fortified cereal beverages remains understudied. Understanding the synergistic effects of these processes on the phytochemical content, aroma profile, and functional properties of cereal‐based beverages fortified with fruits is critical for advancing their development. This study bridges the gap by examining the combined effects of enzymatic hydrolysis and fermentation on sea buckthorn‐based cereal beverages, focusing on their volatile profile, phytochemical content, and functional properties. By exploring these synergistic effects, this work aims to establish a robust foundation for developing next‐generation functional beverages with superior health‐promoting properties.

## 2 Materials and Methods

### 2.1 Raw Materials, Microbial Starter Cultures, and Analytical Reagents

The rice and highland barley used in this study were sourced from a local supermarket in Zhenjiang, Jiangsu Province, China. Sea buckthorn pulp was sourced from Xinjiang Province, China. We sourced *Acetobacter pasteurianus* Ap‐As.1.41 from Yishui Jinrun Biological Technology Co. Ltd. (Yishui, Shandong, China), while *Torulaspora delbrueckii* D1‐3 was isolated from Canadian blueberries in a prior study. Cellulase, α‐amylase, and α‐glucosidase from *Saccharomyces cerevisiae* , p‐nitrophenyl glucopyranoside. The enzyme angiotensin‐converting enzyme (ACE), from rabbit lung, and hippuryl‐L‐histidyl‐L‐leucine (HHL) were obtained from Macklin Biochemical Co. Ltd. (Shanghai, China). Other reagents used were of analytical grade and procured from Sinopharm Chemical Reagent Co. Ltd. (Shanghai, China).

### 2.2 Preparation of Starter Cultures and Beverage

#### 2.2.1 Cultivation of Starter Cultures

The activation of *Acetobacter pasteurianus* (AAB) was performed in RAE broth, comprising 40 g/L glucose, 10 g/L yeast extract, 10 g/L fishmeal peptone, 6.8 g/L disodium hydrogen phosphate dodecahydrate, 1.5 g/L citric acid monohydrate, and 2% absolute ethanol, with 0.01 g of the bacteria in 150 mL of broth. This process occurred over 18 h at 30°C. In contrast, *Torulaspora delbrueckii* D1‐3 (yeast) was activated in YPD yeast peptone dextrose (YPD) broth at 25°C for the same duration. Following activation, both strains underwent two subculturing cycles. They were then centrifuged at 4000 × *g* for 10 min at 4°C. After centrifugation, the microbial cells were washed with a 0.1% NaCl solution and resuspended in sterile distilled water. The optical density (OD) was adjusted to 0.657 for *Acetobacter pasteurianus* and 0.638 for *Torulaspora delbrueckii* D1‐3. The inoculum concentrations were determined using the plate count method, yielding 7.94 log CFU/mL for *Acetobacter pasteurianus* and 6.82 log CFU/mL for *Torulaspora delbrueckii* D1‐3.

#### 2.2.2 Preparation of Beverage

A mixture of rice (150 g) and highland barley (75 g) was soaked in sterile distilled water at a 1:2 ratio (w/v) overnight. After draining, 75 g of sea buckthorn pulp was added and blended (Jujike, Foshan Kelaiya Electrical Appliance Co. Ltd., Foshan City, China) with distilled water at a 1:5 ratio (w/v). Cellulase enzyme (1 g) was dissolved in 100 mL of 0.2 M sodium acetate buffer (pH 6.26). This solution was incorporated into the beverage at concentrations of 3% (v/v) and 5% (v/v), and enzymatic hydrolysis was carried out at 40°C for 4 h. After hydrolysis, the mixture was gelatinized at 80°C for 10 min and then cooled. The pH was adjusted to 5.5, after which 1% (v/v) of each inoculum was added. Fermentation occurred at 30°C for 48 h, with anaerobic conditions for the first 24 h and aerobic conditions for the next 24 h. A control sample remained unfermented.

### 2.3 Determination of pH, Total Soluble Solids (TSS), Reducing Sugar Content (RDS) and Color Parameters

#### 2.3.1 pH, Total Soluble Solids (TSS), Reducing Sugar Content (RDS)

The pH level was measured with a digital pH meter (LIDA Instrument PHS‐3C Precision pH/mV meter). Total soluble solids (TSS) were quantified using an Atago digital refractometer (Japan) and expressed in °Brix. Reducing sugar content was determined via the 3,5‐dinitrosalicylic (DNS) acid method as described by Fischer and Stein (1961). This involved mixing 0.5 mL of DNS reagent with 0.5 mL of SBCB, heating the mixture in a boiling water bath for 5 min, and then cooling it to room temperature. After diluting with 99 mL of water, the absorbance was measured at 540 nm using a UV spectrophotometer.

#### 2.3.2 Assessment of Color Parameters

The CIE color parameters of the samples, including L* (lightness), a* (red‐green axis), and b* (yellow‐blue axis), were determined using a HunterLab ColorQuest XE Spectrophotometer. The total color difference (ΔE) was calculated based on these parameters using the equation:

(1) $$\Delta E=\sqrt{{\left(L_{o}^{*}-L^{*}\right)}^{2}+{\left(a_{o}^{*}-a^{*}\right)}^{2}+{\left(b_{o}^{*}-b^{*}\right)}^{2}}$$

### 2.4 Analysis of Enzyme Activity

Enzyme activity was assessed according to the method described by Zhao et al. (2023). To prepare the crude enzyme solution, SBCB samples were treated as follows: 2 g of sample was mixed with 20 mL of 0.1 M acetate buffer (pH 5.5) and shaken at 200 rpm for 60 min. After centrifugation at 10,000 × g for 10 min at 4°C, the supernatants were collected for analysis. β‐glucosidase activity was determined by mixing 0.1 mL of enzyme extract with 0.9 mL of 200 mM acetate buffer (pH 4.5), followed by incubation at 50°C for 10 min. Then, 1 mL of 5 mM pNPβG was added, and the mixture was incubated for another 10 min at 50°C. The reaction was terminated by adding 1 mL of 1 M Na2CO3, and the release of p‐nitrophenol was measured at 410 nm. One unit of β‐glucosidase activity was defined as the amount of enzyme required to release 1 μmol of p‐nitrophenol per minute under the specified conditions. For laccase activity, the reaction mixture consisted of 100 μL of enzyme extract, 200 μL of ABTS, and 1.7 mL of 0.1 M acetate buffer (pH 5), which was incubated at 37°C for 6 min. The reaction was stopped by adding 500 μL of 0.4 M trichloroacetic acid, and absorbance was measured at 420 nm. One unit of laccase activity was defined as the amount of enzyme required to oxidize 1 mmol of 0.5 mM ABTS per minute.

### 2.5 Determination of Phytochemical Contents and Antioxidant Properties

#### 2.5.1 Total Phenolic Content (TPC), Total Flavonoid Content (TFC), Total Flavonol Content (TFLC), and Total Proanthocyanidin Content (TPAC)

The total phenolic content (TPC) of SBCB was assessed using the Folin–Ciocalteu method, as outlined by Kwaw et al. (2018). The procedure involved adding 2 mL of freshly prepared Folin–Ciocalteu reagent (1:1 v/v) to 0.2 mL of the sample, followed by the addition of 2 mL of sodium carbonate solution (75 g/L). The mixture was vortexed for 1 min and then incubated at 25°C for 40 min. The absorbance was measured at 760 nm using a UV spectrophotometer (UV‐1600, Beijing Rayleigh Analytical Instrument, Beijing, China). The TPC was expressed as milligrams of gallic acid equivalent per 100 g of fresh weight (FW) of the beverage.

The total flavonoid content (TFC) of SBCB was determined according to Kwaw et al. (2018) and expressed as milligrams of rutin equivalent per 100 g of FW. For total flavonol content (TFLC), the method of Kumaran and Karunakaran (2007) was followed. This involved mixing 2 mL of SBCB with equal volumes of 2% AlCl3 and 3 mL of sodium acetate solution (50 g/L), followed by vortexing and incubation at 20°C for 2.5 h. The absorbance was measured at 440 nm, and TFLC was expressed as milligrams of quercetin equivalent (QE) per 100 g of SBCB. The total proanthocyanidin content was assessed using the method described by Boasiako et al. (2024), where 0.5 mL of the sample was mixed with a 4% vanillin‐methanol solution and 1.5 mL of hydrochloric acid. After standing for 15 min, the absorbance was recorded at 500 nm, and the results were expressed as milligrams of catechin equivalent (CE) per 100 g of SBCB.

#### 2.5.2 Measurement of Antioxidant Properties

The ferric reducing antioxidant power (FRAP) assay was performed according to Feng et al. (2019) with slight modifications. The FRAP reagent was freshly prepared by combining 300 mM acetate buffer, 10 mM TPTZ (2,4,6‐tripyridyl‐s‐triazine) in 40 mM HCl, and 20 mM FeCl_3_·6H_2_O in a 10:1:1 (v/v/v) ratio. A 0.2 mL sample was mixed with 3.8 mL of FRAP reagent and incubated at 37°C for 10 min. The absorbance was then measured at 593 nm using a spectrophotometer. The results were expressed as milligrams of Trolox equivalent (TE) per 100 g of SBCB.

The reducing power capacity (RPC) of SBCB was determined according to Kwaw et al. (2018). A 1 mL aliquot of SBCB (diluted 1:100) was mixed with 0.05 mL of 0.01 M HCl, 0.4 mL of 0.02 M potassium ferricyanide, 0.4 mL of 0.02 M FeCl3, and 0.7 mL of distilled water. The mixture was incubated at 37°C in the dark for 30 min, and the absorbance was measured at 720 nm. Results were expressed as milligrams of Trolox equivalent (TE) per 100 g of SBCB.

For DPPH radical scavenging activity (DPPH●‐SA), the method of Li et al. (2021) was followed with slight modifications. A 0.12 mL sample (diluted 1:15) was combined with 4.2 mL of 0.1 mM DPPH solution, vortexed, and incubated at 25°C in the dark for 30 min. Absorbance was measured at 517 nm using a UV spectrophotometer (UV‐1600), and results were expressed as milligrams of TE per 100 g of SBCB.

### 2.6 In Vitro α‐Amylase, α‐Glucosidase and Angiotensin‐Converting‐Enzyme (ACE) Inhibitory Properties Analysis

#### 2.6.1 α‐Amylase Inhibitory Activity Assay

The α‐amylase inhibition activity was evaluated according to Zhang, Li, et al. (2021). A 100 μL sample (250 μg/mL) was combined with 100 μL of 0.2 U/mL α‐amylase and incubated at 25°C for 10 min. Then, 100 μL of 1% soluble starch was added, and the mixture was incubated at 25°C for another 10 min. The reaction was stopped by adding 200 μL of dinitrosalicylic acid reagent (DNS), and the mixture was boiled in a water bath for 5 min. After dilution with distilled water, the absorbance was measured at 540 nm. Distilled water was used instead of the sample in the control. The inhibition activity was calculated using the formula:

(2) $$\text{Inhibition}\;\left(\%\right)=\frac{\text{Acontrol}-\text{Asample}}{\text{Acontrol}}\times 100$$

##### 2.6.1.1 α‐Glucosidase Inhibitory Activity Assay

The α‐glucosidase inhibition assay was performed according to Zhao et al. (2023) with slight modifications. α‐Glucosidase (1 U/mL) and 5 mM p‐nitrophenylglucopyranoside (pNPG) were prepared in 0.1 M phosphate buffer (PBS) at pH 6.8. A 50 μL sample of SBCB (250 μg/mL) was mixed with 100 μL of α‐glucosidase and incubated at 37°C for 20 min. Then, 50 μL of 5 mM pNPG was added, and the mixture was incubated at 37°C for another 30 min. The reaction was stopped by adding 50 μL of 0.1 M sodium carbonate, and absorbance was measured at 405 nm using a UV–Vis spectrophotometer. The inhibitory activity was calculated as follows:

(3) $$\text{Inhibition}\;\left(\%\right)=\left[1-\frac{\left(A0-A1\right)}{\left(A2-A3\right)}\right]\times 100$$

where *A*_0_ represents the absorbance of the reaction mixture, *A*_1_ represents the sample control (without pNPG), *A*_2_ represents the control (without samples), and *A*_3_ represents the blank (phosphate buffer and α‐glucosidase).

#### 2.6.2 Angiotensin‐Converting‐Enzyme (ACE) Inhibitory Properties Analysis

This assay was conducted as described by Obaroakpo et al. (2019) with slight adjustment. ACE solution (25 mU/mL) and 8.3 mM HHL were prepared in 0.1 M sodium borate buffer. A 50 μL aliquot of SBCB solution was combined with 50 μL of ACE solution and incubated at 37°C for 10 min. Subsequently, 150 μL of HHL substrate was added, and the mixture was further incubated at 37°C for 30 min. The reaction was halted by adding 250 μL of 1 M HCl. To extract the liberated hippuric acid (HA), 500 μL of ethyl acetate was introduced. The mixture was then centrifuged at 3000 × *g* at 4°C for 30 min. The ethyl acetate layer was collected into a test tube and evaporated using a water bath at 90°C. The resulting residue (HA) was dissolved in 1.0 mL of sodium borate buffer, and the absorbance was measured at 228 nm using a UV spectrophotometer.

The inhibitory activity was calculated using the following formula:

(4) $$\text{Inhibition}\;\left(\%\right)=\left(1-\frac{\text{Asample}}{\text{Acontrol}}\right)\times 100$$

where Acontrol means absorbance of control (buffer solution) and Asample means Absorbance of SBCB sample.

### 2.7 Determination of Volatile Compounds Using HS‐SPME‐GC–MS

The analysis of volatile components was conducted using a Headspace Solid‐Phase Microextraction (HS‐SPME) system (Agilent Technologies, Santa Clara, CA, USA) following the protocol outlined by Kwaw et al. (2018). A 5 mL sample was mixed with 1.5 g of NaCl in a 15‐mL glass vial, and 10 μL of 2‐octanol (800 μg/L) was added as an internal standard. The mixture was equilibrated at 40°C for 20 min, and a divinylbenzene/carboxen/polydimethylsiloxane (DVB/CAR/PDMS) fiber (50/30 μm) was inserted into the headspace of the vial. The solution was agitated at 2.5 Hz during extraction. After extraction, the fiber was inserted into the injection port of a GC–MS system (Agilent 6890 N–5973 B) equipped with a GC column (60 m × 0.25 mm × 0.25 μm film thickness, Agilent J&W DBWAX). The chromatographic conditions included splitless injection mode with injection and detection temperatures at 250°C, helium as the carrier gas at a flow rate of 1 mL/min, and a temperature program starting at 50°C for 10 min, increasing to 150°C at 6°C/min, then to 200°C at 8°C/min, and held for 7 min. Mass spectrometry was performed with an ion source at 23°C, a quadrupole at 150°C, and electron impact ionization at 70 eV with a scan range of 33–350 amu. Volatile compounds were identified by comparing mass spectra with the National Institute of Standards and Technology (NIST) 17 library database, and semi‐quantification was performed using 2‐octanol as the internal standard. The volatile compounds were calculated using the equation:

(5) $$\mathrm{VC}\;\left(\frac{\mathrm{ng}}{g}\right)=\frac{\text{Peak area ratio}\times 10\mathrm{\mu L}\;\left(\mathrm{ISD}\right)\times 0.8\left(\frac{\mathrm{ng}}{g}\right)\;\left(\mathrm{ISD}\right)}{\text{Equivalent mass of volume used}}$$

### 2.8 Statistical Analysis

Each treatment and assay was carried out in triplicate, and the results are reported as mean values ± standard deviation (SD). Statistical analyses were performed using SPSS Statistics version 26.0 (IBM Corp., Armonk, NY, USA). Differences among treatments were assessed using one‐way analysis of variance (ANOVA), and Duncan's multiple range test was applied for post hoc comparisons, with statistical significance set at *p* < 0.05.

#### 2.8.1 Multivariate and Correlation Analyses

Heat map clustering was used to visualize the volatile profiles of the samples. Principal component analysis (PCA) was carried out to examine relationships among samples and variables, and the Mantel test was applied to assess correlations between antioxidant properties, phytochemical contents, physicochemical parameters, color indices, and enzyme activity. All analyses were generated using the Cloudtutu software.

#### 2.8.2 Mantle Test

The Mantel test was applied to assess correlations between biological activities (antioxidant, antidiabetic, and ACE inhibitory properties) and measured parameters including phytochemical composition, physicochemical indices, color values, and enzyme activity. This analysis was performed using cloudtutu.

## 3 Results and Discussion

### 3.1 Effect of Enzyme Treatment and Fermentation on the Physicochemical Properties and Enzyme Activity of Sea Buckthorn‐Based Cereal Beverage

The physicochemical properties and enzyme activity of unfermented and enzyme‐assisted fermented SBCB are presented in Table 1. Fermentation significantly affected the pH values, with a more pronounced decrease observed in enzyme‐assisted fermented samples, especially in F5% (fermented SBCB treated with 5% enzyme). This indicates that enzyme pretreatment, particularly at higher concentrations, enhances the breakdown of complex carbohydrates into simple sugars, which are then readily utilized by fermenting microorganisms to produce organic acids, as shown in Table 1. The total soluble solids (TSS) reduced drastically after fermentation compared to the control (WEWF, unfermented and non‐enzyme‐treated SBCB) and enzyme‐treated only samples WF3% (unfermented, 3% enzyme‐treated SBCB) and WF5% (unfermented, 5% enzyme‐treated SBCB). Although a slight increase in TSS was observed in WF3% and WF5% compared to the control, it was not statistically significant. Enzymatic hydrolysis significantly increased the reducing sugar content (RDS), with increments of 91% and 101.22% observed in the WF3% and WF5% samples, respectively, against the control. These findings conform with Lao et al. (2020), who reported increased reducing sugars following enzymatic hydrolysis. In contrast, fermentation led to a reduction in reducing sugar content, as observed in the WEF, F3%, and F5% samples. The positive correlation between TSS and RDS suggests that as TSS decreases, RDS also decreases, likely due to the conversion of sugars to organic acids by microorganisms, resulting in a corresponding decrease in pH. This aligns with the study of Lao et al. (2020), which reported decreases in pH and TSS in enzyme‐assisted fermented *Cordyceps militaris* beverages. Color measurement is paramount in the food industry, as it significantly influences consumer perception and acceptance. The color parameters L*, a*, b*, and ∆E exhibited the following ranges: L* values from 65.52 ± 0.09 (WEWF) to 48.78 ± 0.10 (F5%); a* values from 14.69 ± 0.04 (WF5%) to 6.95 ± 0.34 (F5%); b* values from 49.51 ± 0.10 (WEWF) to 31.80 ± 0.68 (F5%); and ∆E values from 32.15 ± 0.13 (WF3%) to 0.39 ± 0.18 (WF5%). The lightness (L*) decreased in all treated samples compared to the control, with fermentation alone displaying higher L* values, while the combination of enzyme treatment and fermentation resulted in a darker beverage, particularly evident in F5%. The redness values (a*) increased in enzyme‐only and fermentation‐only treatments, while a significant decrease was noted in the enzyme‐assisted fermented treatments. All treated samples exhibited a decrease in yellowness (b*) compared to the control, with the F5% sample displaying the most pronounced reduction. These findings suggest that pretreatment may have led to the breakdown of color pigments, contributing to the observed changes in color parameters. Microbial enzymatic activity can enhance the bioactive components of fermented foods (Sharma et al. 2020). The results of β‐glucosidase and laccase activities are presented in Table 1. β‐Glucosidase activity was significantly higher in samples treated with the higher enzyme concentration (WF5%), as well as in the fermentation‐only and enzyme‐assisted fermentation treatments. Laccase activity was elevated in the treated samples relative to the control. The F5% treatment demonstrated the highest activities for both β‐glucosidase (505.98 ± 7.96 U/g) and laccase (2986.67 ± 66.67 U/g). Overall, the combination of enzyme treatment and fermentation resulted in higher enzyme activities, surpassing the levels observed in the other treatments and the control.

**TABLE 1:** Effect of enzyme treatment and fermentation on the physicochemical properties and enzyme activity of sea buckthorn‐based cereal beverage.

Parameter	WEWF	WF3%	WF5%	WEF	F3%	F5%
Physicochemical properties						
pH	5.50 ± 0.00^a^	5.50 ± 0.00^a^	5.50 ± 0.00^a^	4.76 ± 0.01^b^	3.24 ± 0.01^c^	2.69 ± 0.11^d^
TSS (°Brix)	10.00 ± 0.00^a^	10.33 ± 0.29^a^	10.50 ± 0.50^a^	6.33 ± 0.29^c^	6.83 ± 0.29^c^	7.83 ± 0.29^b^
Reducing sugar (g/100 g)	4.11 ± 0.09e	7.85 ± 0.04b	8.27 ± 0.04a	1.52 ± 0.04f	6.25 ± 0.03c	5.51 ± 0.03d
L*	65.52 ± 0.09^a^	61.25 ± 0.06^c^	60.10 ± 0.04^d^	62.40 ± 0.01^b^	56.82 ± 0.16^e^	48.78 ± 0.10^f^
a*	14.51 ± 0.03^a^	14.61 ± 0.03^a^	14.69 ± 0.04^a^	14.63 ± 0.03^a^	12.86 ± 0.07^b^	6.95 ± 0.34^c^
b*	49.51 ± 0.10^a^	45.22 ± 0.13^c^	45.14 ± 0.17^c^	48.83 ± 0.10^b^	44.44 ± 0.29^d^	31.80 ± 0.68^e^
∆E	0.88 ± 0.12^e^	32.15 ± 0.13^a^	0.39 ± 0.18^e^	4.73 ± 0.10^c^	3.58 ± 0.31^d^	18.75 ± 0.57^b^
Phytochemical content						
TPC (mg GAE/100 g)	147.41 ± 0.34^f^	167.61 ± 0.34^e^	268.56 ± 0.57^c^	187.06 ± 1.16^d^	331.58 ± 0.45^a^	317.16 ± 0.45^b^
TFC (mgRE/100 g)	115.03 ± 0.11^f^	154.85 ± 0.95^d^	141.09 ± 0.40^e^	212.82 ± 0.39^c^	281.01 ± 0.29^b^	319.49 ± 0.49^a^
TFLC (mgQE/100 g)	18.99 ± 0.04^f^	41.84 ± 0.05^c^	40.35 ± 0.11^d^	20.89 ± 0.07^e^	46.95 ± 0.12^a^	43.90 ± 0.11^b^
TPAC (mgCE/100 g)	1614.29 ± 4.39^e^	2069.70 ± 3.32^c^	1621.00 ± 3.21^e^	2040.94 ± 9.25^d^	2290.22 ± 3.21^b^	2555.80 ± 4.39^a^
Enzyme activity						
β‐glucosidase activity (U/g)	227.82 ± 3.98^e^	227.82 ± 10.53^e^	328.97 ± 6.90^c^	292.18 ± 3.98^d^	439.31 ± 0.00^b^	505.98 ± 7.96^a^
Laccase activity (U/g)	1675.56 ± 19.25^e^	2164.44 ± 38.49^c^	1964.44 ± 38.49^d^	2142.22 ± 38.49^c^	2342.22 ± 38.49^eb^	2986.67 ± 66.67^a^

*Note:* WEWF (Control) represents untreated SBCB, WF3% represents unfermented, 3% enzyme‐treated SBCB, WF5% represents unfermented, 5% enzyme‐treated SBCB, WEF represents non‐enzyme‐treated, fermented SBCB, F3% represents fermented, 3% enzyme‐treated SBCB, F5% represents fermented, 5% enzyme‐treated SBCB, SBCB represents sea buckthorn–based cereal beverage. Values are expressed as mean ± standard deviation (SD), *n* = 3. Means with different letters indicate significant differences at *p* < 0.05. TPC means total phenolic content, TFC means total flavonoid content, TFLC, means total flavonol content, TPAC means total proanthocyanidin content.

### 3.2 Effect of Enzyme Treatment and Fermentation on Phytochemical Contents of Sea Buckthorn‐Based Cereal Beverage

Plant‐derived phytochemicals, including phenolic acids, flavonoids, flavonols, and proanthocyanidins, are associated with antioxidant, antidiabetic, ACE inhibitory, anti‐inflammatory, anticancer, and antimicrobial properties (Kumar et al. 2023). Enzyme‐assisted fermentation increased all measured phytochemical content compared to the control. Enzyme‐assisted fermentation significantly enhanced the phytochemical content compared to the control. The most pronounced changes occurred in F3%, where TPC rose by 124.94% and TFLC by 147.24%, and in F5%, where TFC increased by 177.74% and TPAC by 58.32%. These outcomes indicate that an enzymatic pretreatment that opens the plant matrix, followed by targeted fermentation, synergistically elevates extractable phenolic constituents. Milić et al. (2023) similarly documented increased phenolic content through combined enzymatic and microbial fermentation of spent ground coffee. The observed increases in phytochemicals likely reflect enzyme‐mediated hydrolysis under acidic conditions by *A. pasteurianus* and *T. delbrueckii* D1‐3, whose β‐glucosidase, esterase, cellulase, glucanase, xylanase, pectinase, and laccase activities degrade cell walls and cleave glucosidic linkages to liberate bound phenolics (Neffe‐Skocińska et al. 2023; Phung et al. 2023). This finding is consistent with previous research, such as Phung et al. (2023), which highlighted the synergistic effects of microbial enzymes in enhancing phenolic content. Our results also corroborate the work of Zhang, Zhang, et al. (2021), who demonstrated that enzymatic hydrolysis and enzyme‐assisted fermentation can increase TPC and TFC, with enzyme‐assisted fermentation yielding higher values. A plausible mechanism is β‐glucosidase‐mediated deglycosylation during mixed fermentation with yeast and acetic acid bacteria. Yeasts, including Torulaspora delbrueckii, possess β‐glucosidases that hydrolyze glucosidic linkages in phenolic glycosides, releasing aglycones. The liberation of aglycones is expected to elevate TPC and antioxidant properties, thereby altering the phenolic composition of the product (Zhang, Zhang, et al. 2021). In a comparable system without enzymatic pretreatment, coffee fermentation increased TPC by ~1.8–2.0‐fold and TFC by ~2.1–2.6‐fold within 24 h, consistent with microbial glycosidases operating in an acidifying environment (Kim et al. 2025). Furthermore, Milić et al. (2023) reported a 67% rise in TPC in enzyme‐assisted fermentation of spent coffee grounds. By contrast, our enzyme‐assisted fermentation increased extractable phenolics beyond these reported upper bounds, namely 2.6‐fold in non‐enzymatic systems and 67% in enzyme‐assisted fermentation systems.

### 3.3 Effect of Enzyme Treatment and Fermentation on Bioactivity of Sea Buckthorn‐Based Cereal Beverage

#### 3.3.1 Antioxidant Properties

**FIGURE 1:**
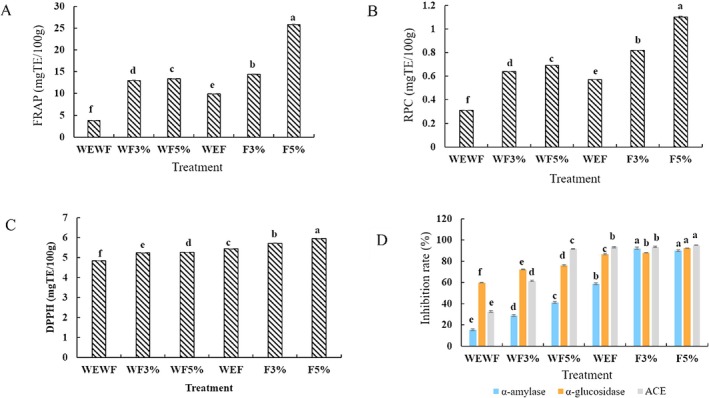
Effect of enzyme‐assisted fermentation on the bioactivity of sea buckthorn‐based cereal beverage. Antioxidant properties measured by FRAP (A), RPC (B), and DPPH (C), α‐amylase, α‐glucosidase, and ACE inhibitory activity (D). WEWF (Control) represents untreated SBCB, WF3% represents unfermented, 3% enzyme‐treated SBCB, WF5% represents unfermented, 5% enzyme‐treated SBCB, WEF represents non‐enzyme‐treated, fermented SBCB, F3% represents fermented, 3% enzyme‐treated SBCB, F5% represents fermented, 5% enzyme‐treated SBCB, SBCB represents sea buckthorn–based cereal beverage. Data are shown as mean ± standard deviation (SD), *n* = 3. Different letters denote significant differences at *p* < 0.05.

Antioxidants can neutralize free radicals through various mechanisms, including metal chelation, free radical scavenging, and enzyme inhibition, thereby preventing oxidative damage. To evaluate the antioxidant capacity of SBCB, several methods have been utilized. Our analysis of antioxidant properties using FRAP, RPC, and DPPH assays (Figure 1A–C) showed that enzyme treatment significantly boosted SBCB's antioxidant potential, especially in FRAP and RPC. Moreover, combining enzyme treatment with fermentation further enhanced antioxidant properties across all three assays, outperforming both the control and fermentation‐alone treatments. Notably, the F5% treatment yielded substantial increases in antioxidant activity, with 6.78‐fold, 3.55‐fold, and 1.22‐fold enhancements in FRAP, RPC, and DPPH, respectively. Zhang, Zhang, et al. (2021) observed a similar enhancement in antioxidant properties with enzyme‐assisted fermentation of quinoa. Milić et al. (2023) also reported increased antioxidant activity following combined enzymatic and microbial fermentation of spent ground coffee. Additionally, previous studies have demonstrated elevated antioxidant properties in beverages fermented with *Acetobacter pasteurianus* and *Torulaspora delbrueckii* (Freire et al. 2017; Zhu et al. 2022).

#### 3.3.2 In Vitro Antidiabetic and Angiotensin‐Converting Enzyme (ACE) Inhibitory Properties

Our analysis of in vitro antidiabetic and antihypertensive properties, as depicted in Figure 1D and Table 2, revealed that both fermentation alone and enzyme‐assisted fermentation significantly improved α‐amylase, α‐glucosidase, and ACE inhibitory activities in SBCB compared to enzyme treatment alone and the control. Notably, F3% exhibited the highest α‐amylase inhibitory activity (92.22% ± 0.92%), whereas F5% showed the highest α‐glucosidase (92.38% ± 0.15%) and ACE inhibitory (95.24% ± 0.28%) activities. These results demonstrate a synergistic effect between enzyme treatment and fermentation in enhancing SBCB's bioactivity. The superior inhibitory activities observed in F3% and F5% can be attributed to the increased concentration of bioactive compounds, highlighting their potential as antidiabetic and antihypertensive agents. Our findings align with previous studies, such as Tang et al. (2024), who reported enhanced α‐amylase and α‐glucosidase inhibitory activities during lactic acid fermentation of passion fruit peels, and Liu et al. (2022b), who observed increased ACE inhibitory potential in fermented tea. These results provide a strong foundation for further investigation into enzyme‐assisted fermentation as a strategy for developing functional beverages with targeted health benefits.

**TABLE 2:** In vitro antidiabetic and angiotensin‐converting enzyme (ACE) inhibitory properties.

Enzyme activity	WEWF	WF3%	W5%	WEF	F3%	F5%
α‐Amylase inhibitory properties (%)	15.56 ± 0.92^e^	28.89 ± 0.92^d^	41.11 ± 0.92^c^	58.89 ± 0.92^b^	92.22 ± 0.92^a^	90.00 ± 0.92^a^
α‐Glucosidase inhibitory properties (%)	59.82 ± 0.29^f^	72.29 ± 0.44^e^	76.34 ± 0.45^d^	86.80 ± 0.51^c^	88.07 ± 0.08^b^	92.38 ± 0.15^a^
ACE inhibitory properties (%)	32.68 ± 0.75^e^	61.58 ± 0.48^d^	91.63 ± 0.49^c^	93.27 ± 0.57^b^	93.60 ± 0.49^b^	95.24 ± 0.28^a^

*Note:* WEWF (Control) represents untreated SBCB, WF3% represents unfermented, 3% enzyme‐treated SBCB, WF5% represents unfermented, 5% enzyme‐treated SBCB, WEF represents non‐enzyme‐treated, fermented SBCB, F3% represents fermented, 3% enzyme‐treated SBCB, F5% represents fermented, 5% enzyme‐treated SBCB, SBCB represents sea buckthorn–based cereal beverage. Values are expressed as mean ± standard deviation (SD), *n* = 3. Means with different letters indicate significant differences at *p* < 0.05.

### 3.4 Effect of Enzyme Treatment and Fermentation on Volatile Profile of Sea Buckthorn‐Based Cereal Beverage

The quantification of volatile compounds is presented in Table 3, with corresponding chromatograms shown in Figure S1. Our analysis identified a total of 66 volatile compounds, which comprised 14 alcohols, 11 acids, 24 esters, 6 ketones, 8 aldehydes, and 3 other compounds. Among the alcohols, 3‐methyl‐1‐butanol was predominant in SBCB, showing a 35.48‐fold increase in F3% compared to the control. Phenylethyl alcohol was found in all samples except WF5%, with higher levels in fermented samples, particularly WEF, and is known to impart a floral note due to yeast activity (Phung et al. 2023). 2‐Methyl propan‐1‐ol was detected exclusively in fermented samples, with higher concentrations in F3% and F5%. Among the acids, 3‐methylbutanoic acid and hexanoic acid were the most abundant. Acetic acid was present in all samples except the control, with high concentrations in F3% (554.48 ± 0.79 ng/100 g) and F5% (693.93 ± 1.42 ng/100 g). Sorbic acid was found only in samples with 5% enzyme treatments (WF5% and F5%), while benzoic and butanoic acids were detected only in F3% and F5%. Sorbic and benzoic acids, used as preservatives in various foods, can serve a similar function in SBCB (Javanmardi et al. 2015). 2‐Methyl propanoic acid and hexadecanoic acid were found exclusively in fermented samples, with the highest concentrations in F5% and WEF, respectively, likely due to the activity of fermenting microorganisms.

**TABLE 3:** Quantification of aroma components of unfermented and enzyme‐assisted fermented sea buckthorn‐based cereal beverage.

Volatiles	S/N	Compound name	Concentration (ng/100 g)					
			WEWF	WF3%	WF5%	WEFF	F3%	F5%
Alcohols	AL1	Hexan‐1‐ol	7.64 ± 0.15^a^	4.44 ± 0.32^b^	7.39 ± 0.21^a^	nd	nd	nd
	AL2	3‐Methy butan‐1‐ol	7.82 ± 0.29^e^	13.75 ± 0.42^d^	15.10 ± 0.27^d^	19.26 ± 0.13^c^	277.42 ± 2.64^a^	165.31 ± 3.49^b^
	AL3	Phenylethyl alcohol	4.05 ± 0.15^d^	3.77 ± 0.06^d^	nd	88.56 ± 0.85^a^	46.02 ± 1.26^c^	63.47 ± 1.83^b^
	AL4	1‐Pentanol	2.86 ± 0.06^b^	3.06 ± 0.20^b^	5.47 ± 0.39^a^	nd	nd	nd
	AL5	Cycloheptanol	0.66 ± 0.03^a^	nd	nd	nd	nd	nd
	AL6	1‐Penten‐3‐ol	nd	0.73 ± 0.03^a^	nd	nd	nd	nd
	AL7	1‐Octen‐3‐ol	nd	nd	2.31 ± 0.03^a^	nd	nd	nd
	AL8	1‐Dodecanol	nd	nd	1.49 ± 0.02^a^	nd	nd	nd
	AL9	2,3‐Butanediol	nd	nd	nd	12.69 ± 0.21^b^	26.63 ± 0.39^a^	nd
	AL10	4‐Ethyl‐phenol	nd	nd	nd	7.99 ± 0.17^a^	nd	nd
	AL11	Phenol	nd	nd	nd	3.75 ± 0.06^a^	nd	nd
	AL12	2‐Methyl propan‐1‐ol	nd	nd	nd	3.49 ± 0.11^c^	64.52 ± 0.43^a^	56.24 ± 0.66^b^
	AL13	3‐Methyl‐2‐heptanol	nd	nd	nd	2.58 ± 0.18^a^	nd	nd
	AL14	2,4‐Di‐tert‐butylphenol	nd	nd	nd	nd	1.69 ± 0.02^a^	nd
		Total alcohols (TAL)	23.04 ± 0.38^e^	25.75 ± 0.66^e^	31.76 ± 0.40^d^	138.33 ± 1.42^c^	416.28 ± 2.95^a^	285.02 ± 5.69^b^
Acids	AC1	3‐Methylbutanoic acid	49.67 ± 0.63^d^	51.22 ± 0.90^d^	45.06 ± 0.70^e^	104.00 ± 1.52^b^	80.84 ± 0.24^c^	193.32 ± 0.93^a^
	AC2	Hexanoic acid	10.76 ± 0.23^d^	12.94 ± 0.97^c^	12.79 ± 0.29^c^	20.41 ± 0.26^a^	11.05 ± 0.36^d^	19.06 ± 0.20^b^
	AC3	Octanoic acid	0.84 ± 0.03^d^	0.85 ± 0.03^c^	1.74 ± 0.02^c^	nd	nd	nd
	AC4	Acetic acid	nd	125.26 ± 1.09^d^	90.10 ± 0.79^e^	189.39 ± 0.68^c^	554.48 ± 0.79^b^	693.93 ± 1.42^a^
	AC5	Pentanoic acid	nd	0.83 ± 0.02^a^	nd	nd	nd	nd
	AC6	Sorbic Acid	nd	nd	1.06 ± 0.02^b^	nd	nd	3.15 ± 0.05^a^
	AC7	Heptanoic acid	nd	nd	0.92 ± 0.01^a^	nd	nd	nd
	AC8	2‐Methyl propanoic acid	nd	nd	nd	19.69 ± 0.40^b^	7.89 ± 0.09^c^	50.70 ± 0.36^a^
	AC9	Hexadecanoic acid	nd	nd	nd	6.01 ± 0.13^a^	2.92 ± 0.03^c^	3.19 ± 0.11^b^
	AC10	Benzoic acid	nd	nd	nd	nd	4.55 ± 0.23^a^	3.06 ± 0.08^b^
	AC11	Butanoic acid	nd	nd	nd	nd	8.88 ± 0.16^a^	2.25 ± 0.14^b^
		Total acids (TA)	61.27 ± 0.44^f^	191.11 ± 2.05^d^	151.67 ± 1.77^e^	339.49 ± 1.61^c^	670.52 ± 1.65^b^	968.66 ± 3.03^a^
Esters	ES1	1‐Butanol, 3‐methyl‐, benzoate	23.07 ± 0.63^b^	17.57 ± 0.35^c^	26.69 ± 0.37^a^	nd	nd	nd
	ES2	Ethyl hexanoate	19.69 ± 0.26^b^	11.44 ± 0.32^c^	40.72 ± 0.60^a^	5.65 ± 0.32^d^	nd	nd
	ES3	Isopentyl hexanoate	17.67 ± 0.42^b^	4.80 ± 0.27^c^	20.27 ± 0.28^a^	nd	nd	nd
	ES4	Ethyl octanoate	16.27 ± 0.20^b^	nd	nd	1038.47 ± 1.88^a^	nd	nd
	ES5	Ammonium acetate	7.27 ± 0.22^a^	nd	nd	nd	nd	nd
	ES6	Ethyl pentadec‐9‐enoate	4.63 ± 0.25^a^	4.27 ± 0.09^b^	nd	nd	nd	nd
	ES7	Ethyl 3‐hydroxy‐3‐methylbutanoate	3.45 ± 0.25^a^	nd	nd	nd	1.81 ± 0.07^b^	1.71 ± 0.02^b^
	ES8	Ethyl 3‐methylbutanoate	3.20 ± 0.03^b^	2.87 ± 0.08^c^	9.89 ± 0.22^a^	nd	nd	nd
	ES9	8‐Methylnonanoic acid, ethyl ester	3.09 ± 0.17^b^	nd	nd	4.57 ± 0.24^a^	nd	nd
	ES10	2‐Phenylethyl 3‐methylbutanoate	2.66 ± 0.22^a^	nd	nd	nd	nd	nd
	ES11	3‐Methylbutyl 2‐methylbutanoate	2.28 ± 0.15^b^	nd	3.85 ± 0.17^a^	nd	nd	nd
	ES12	Isobutyl isovalerate	2.53 ± 0.43^b^	nd	4.85 ± 0.19^a^	nd	nd	nd
	ES13	Ethyl 2‐methylbutanoate	0.87 ± 0.01^b^	0.76 ± 0.04^b^	2.74 ± 0.16^a^	nd	nd	nd
	ES14	Hexanoic acid, 2‐methylpropyl ester	0.82 ± 0.01^a^	nd	nd	nd	nd	nd
	ES15	Phthalic acid, 4,4‐dimethylpent‐2‐yl butyl ester	0.82 ± 0.02^a^	nd	nd	nd	nd	nd
	ES16	3‐Methylbutyl 3‐methylbutanoate	nd	28.71 ± 0.30^c^	107.84 ± 0.34^a^	52.70 ± 0.34^b^	13.57 ± 0.17^d^	13.83 ± 0.11^a^
	ES17	Ethyl decanoate	nd	0.88 ± 0.02^b^	3.52 ± 0.22^a^	nd	nd	nd
	ES18	Pentanoic acid 1‐methylpropyl ester	nd	0.78 ± 0.02^a^	nd	nd	nd	nd
	ES19	Ethyl benzoate	nd	nd	7.24 ± 0.16^a^	nd	1.72 ± 0.03^b^	nd
	ES20	6‐Methylhept‐4‐en‐1‐yl 3‐methylbutanoate	nd	nd	2.60 ± 0.26^a^	nd	nd	nd
	ES21	2‐Phenylethyl 2‐methylbutanoate	nd	nd	2.69 ± 0.28^a^	nd	nd	nd
	ES22	2‐Phenylethyl acetate	nd	nd	nd	5.89 ± 0.14^a^	nd	nd
	ES23	1‐Butanol, 3‐methyl‐, acetate	nd	nd	nd	nd	10.00 ± 0.14^b^	11.84 ± 0.28^a^
	ES24	Isobutyl acetate	nd	nd	nd	nd	2.29 ± 0.18^b^	2.91 ± 0.08^a^
		Total esters (TES)	108.33 ± 3.15^c^	72.07 ± 1.23^d^	232.92 ± 2.94^b^	1107.29 ± 2.87^a^	29.39 ± 0.43^e^	30.29 ± 0.48^e^
Ketones	KT1	5‐Hepten‐2‐one, 6‐methyl—	4.40 ± 0.29^c^	5.71 ± 0.24^b^	11.44 ± 0.36^a^	nd	3.70 ± 0.23^d^	5.68 ± 0.21^b^
	KT2	Pantolactone	1.32 ± 0.20^a^	nd	nd	nd	nd	nd
	KT3	Acetoin	nd	1.53 ± 0.02^d^	nd	217.81 ± 0.24^a^	7.53 ± 0.21^c^	17.41 ± 0.35^b^
	KT4	1‐Hydroxy‐2‐propanone	nd	nd	nd	nd	5.36 ± 0.28^a^	2.52 ± 0.32^b^
	KT5	3‐Octanone	nd	nd	nd	nd	1.73 ± 0.04^b^	3.28 ± 0.15^a^
	KT6	2‐Heptanone	nd	nd	nd	nd	nd	1.90 ± 0.03^a^
		Total ketones (TK)	5.71 ± 0.50^f^	7.24 ± 0.25^e^	11.44 ± 0.36^d^	217.81 ± 0.24^a^	18.33 ± 0.62^c^	30.80 ± 0.95^b^
Aldehydes	ad1	Nonanal	0.83 ± 0.02^c^	0.94 ± 0.02^b^	1.71 ± 0.02^a^	nd	nd	nd
	ad2	Hexanal	2.28 ± 0.17^e^	4.61 ± 0.30^d^	13.44 ± 0.30^b^	nd	5.83 ± 0.08^c^	17.49 ± 0.34^a^
	ad3	5‐Methyl hexanal	nd	nd	2.65 ± 0.12^a^	nd	nd	nd
	ad4	2‐Pentenal	nd	nd	0.86 ± 0.02^a^	nd	nd	nd
	ad5	Benzeneacetaldehyde	nd	nd	0.94 ± 0.01^a^	nd	nd	nd
	ad6	Benzaldehyde	nd	nd	nd	nd	3.32 ± 0.21^b^	47.68 ± 0.41^a^
	ad7	2,4‐Dimethyl benzaldehyde	nd	nd	nd	nd	2.61 ± 0.21^a^	nd
	ad8	2‐Heptenal	nd	nd	nd	nd	2.25 ± 0.20^b^	4.79 ± 0.13^a^
		Total aldehydes (TAD)	3.11 ± 0.19^d^	5.55 ± 0.32^d^	19.60 ± 0.43^b^	nd	14.01 ± 0.57^c^	69.95 ± 0.18^a^
Others	OT1	4‐Octenoic acid, ethyl ether	4.44 ± 0.11^b^	2.32 ± 0.14^d^	7.71 ± 0.18^a^	2.84 ± 0.07^c^	nd	nd
	OT2	Oxime‐, methoxy‐phenyl‐_	nd	10.51 ± 0.18^b^	nd	56.79 ± 0.30^a^	nd	nd
	OT3	2‐Pentyl‐furan	nd	nd	1.45 ± 0.02^a^	nd	nd	nd
		Total others (TO)	4.44 ± 0.11^d^	12.83 ± 0.32^b^	9.16 ± 0.19^c^	59.64 ± 0.28^a^	nd	nd
		Total volatiles	205.90 ± 4.18^f^	314.55 ± 4.80^e^	456.55 ± 5.81^d^	1862.56 ± 5.89^a^	1146.55 ± 2.98^c^	1384.72 ± 10.14^b^

*Note:* WEWF (Control) represents untreated SBCB, WF3% represents unfermented, 3% enzyme‐treated SBCB, WF5% represents unfermented, 5% enzyme‐treated SBCB, WEF represents non‐enzyme‐treated, fermented SBCB, F3% represents fermented, 3% enzyme‐treated SBCB, F5% represents fermented, 5% enzyme‐treated SBCB, SBCB represents sea buckthorn–based cereal beverage. Values are expressed as mean ± standard deviation (SD), *n* = 3. Means with different letters are significantly different (*p* < 0.05).

Esters, known for imparting fruity aromas, were significantly influenced by enzyme hydrolysis and fermentation. Fermentation alone resulted in higher ester concentrations, with ethyl octanoate contributing to 93.78% of the total esters in this sample. Ethyl octanoate is characterized by apricot, brandy, fat, floral, and pineapple aromas (Kwaw et al. 2017). Ester concentrations were extremely low in enzyme‐assisted fermented samples in comparison with other treatments and the control. possibly suggesting hydrolysis of esters to the corresponding high concentration of alcohols and acids (Figure S2). Notably, 1‐butanol, 3‐methyl‐, benzoate was only detected in the control and enzyme‐treated samples, but not in the fermented samples. This absence may be due to hydrolysis of the compound into its corresponding alcohol and acid during fermentation (Park et al. 2009). Esterase activity from fermenting microorganisms might account for this hydrolysis (Sumby et al. 2013), explaining the high concentrations of 3‐methylbutanoic acid in fermented samples and the presence of benzoic acid in F3% and F5%. The absence of compounds like ethyl 3‐methylbutanoate, 2‐phenylethyl 3‐methylbutanoate, isobutyl isovalerate, and 6‐methylhept‐4‐en‐1‐yl 3‐methylbutanoate in fermented samples might contribute to the high concentration of 3‐methylbutanoic acid. High levels of 3‐methylbutan‐1‐ol in fermented samples, particularly F3% and F5%, could explain the absence of 3‐methylbutyl 2‐methylbutanoate. The absence of isobutyl isovalerate in fermented samples might be linked to the high concentration of 2‐methyl propan‐1‐ol found only in these samples. Although lower ester levels are expected to lessen fruity/floral impact and may reduce acceptability, our samples showed higher 3‐methyl‐1‐butanol and increased phenylethyl alcohol, which contribute fruity and floral notes that mitigate the impact of reduced esters and support a balanced aroma profile (Liu, Li, et al. 2022a; Romano et al. 2022; Blesi 2023).

Among the ketones, acetoin was detected only in fermented samples and WF3%, with WEF showing the highest concentration (217.81 ± 0.24 ng/100 g). 1‐Hydroxy‐2‐propanone and 3‐octanone were observed only in enzyme‐assisted fermented samples. Nonanal was found in all treatments except the fermented samples, while hexanal was detected in all samples except WEF. Benzaldehyde and 2‐heptenal were detected only in enzyme‐assisted fermented samples, with higher concentrations in F5%. Benzaldehyde, accounting for 68.16% of the total aldehydes in F5%, imparts bitter almond, burnt sugar, and cherry notes to beverages (Kwaw et al. 2017).

#### 3.4.1 Hierarchical Clustering Analysis and Heat Map Visualization of the Volatile Profiles of SBCB

**FIGURE 2:**
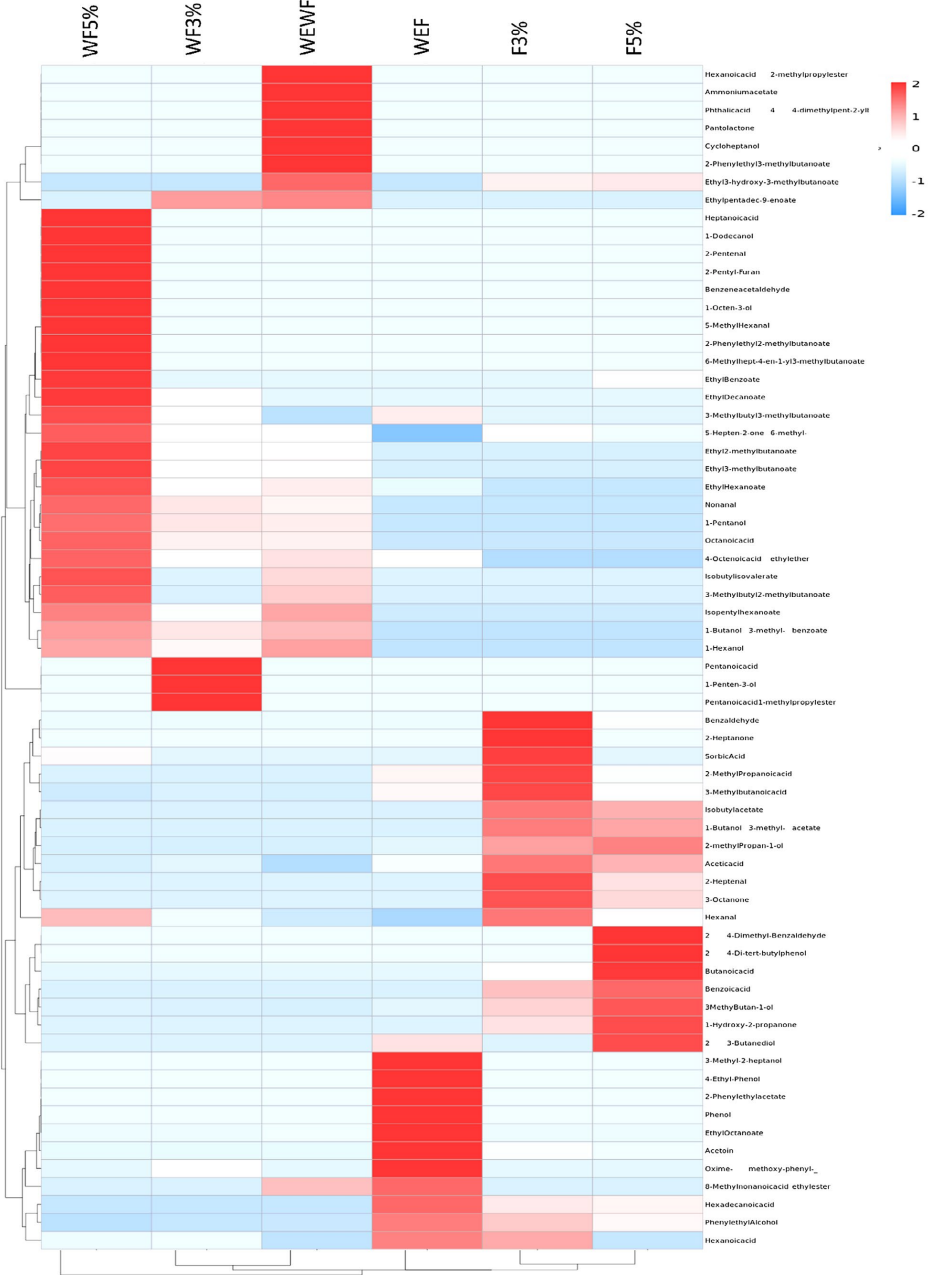
Heat map clustering of volatile profile of enzyme‐assisted fermented sea buckthorn‐based cereal beverage. WEWF (control) represents untreated SBCB, WF3% represents unfermented, 3% enzyme‐treated SBCB, WF5% represents unfermented, 5% enzyme‐treated SBCB, WEF represents non‐enzyme‐treated, fermented SBCB, F3% represents fermented, 3% enzyme‐treated SBCB, F5% represents fermented, 5% enzyme‐treated SBCB, SBCB represents sea buckthorn–based cereal beverage.

The heat map in Figure 2 illustrates the relative abundance of various volatile compounds across different SBCB samples subjected to different treatments. The color gradient from red to blue indicates high to low abundance, respectively. Hierarchical clustering revealed a close relationship between the F3% and F5% samples, attributed to their high concentrations of acids and alcohols, as depicted in Figure S2, along with the presence of isobutyl acetate, 1‐butanol, 3‐methyl‐acetate, 1‐hydroxy‐2‐propanone, 3‐octanone, and 2‐heptenal, indicated in red in Figure 2. The similarity between these samples suggests that the synergy between enzymatic treatment and fermentation influences the aroma profile of SBCB. These samples are distinctly separated from other samples but remain relatively close to WEF. Fermented samples cluster separately from unfermented samples, underscoring the impact of fermentation on the metabolic composition of SBCB. This clustering explains the similarity among fermented samples, particularly in terms of high alcohol and acid content, resulting from the conversion of sugars by fermenting microorganisms. WEWF and WF3% cluster together due to the presence of ethyl pentadec‐9‐enoate. In contrast, WF5% forms its own cluster because of unique volatiles such as 1‐octen‐3‐ol, 1‐dodecanol, heptanoic acid, 2‐phenylethyl 2‐methylbutanoate, 5‐methyl hexanal, 2‐pentenal, and benzeneacetaldehyde, which are absent in other treatments and the control. However, WF5% shows some proximity to the WEWF and WF3% clusters.

#### 3.4.2 Principal Component Analysis (PCA)

**FIGURE 3:**
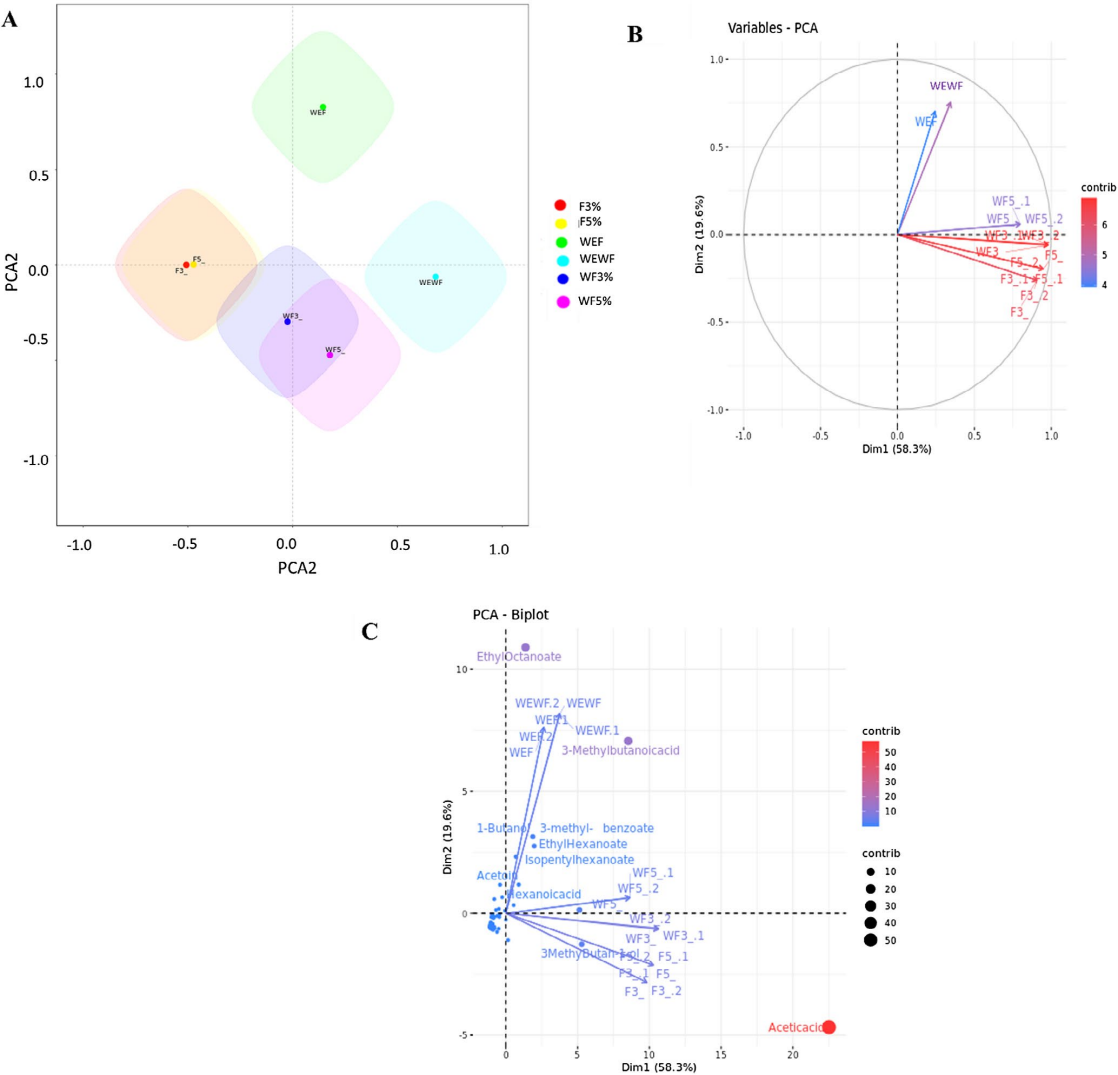
Principal component analysis, PCA (A), Variables PCA (B), PCA biplot of volatile profile of enzyme‐assisted fermented sea buckthorn‐based cereal beverage. WEWF (Control) represents untreated SBCB, WF3% represents unfermented, 3% enzyme‐treated SBCB, WF5% represents unfermented, 5% enzyme‐treated SBCB, WEF represents non‐enzyme‐treated, fermented SBCB, F3% represents fermented, 3% enzyme‐treated SBCB, F5% represents fermented, 5% enzyme‐treated SBCB, SBCB represents sea buckthorn–based cereal beverage. In (B) and (C) denotes %.

Principal component analysis (PCA) of the sea buckthorn‐based cereal beverage (SBCB) samples revealed distinct biochemical differences resulting from fermentation and enzyme treatments, as illustrated in Figure 3A–C. This plot highlights distinct profiles between fermented and unfermented samples. WEWF and WEF are positioned away from other samples, indicating distinct profiles. The high concentration of ethyl octanoate in this sample might be responsible for the clear separation of WEF while low concentrations of acids and alcohols, as well as the absence of some volatiles, might be responsible for the separation observed in the control. WF3% overlaps with WF5% indicating similar profiles influenced by enzyme treatment without fermentation. Similarly, F3% and F5% overlap, showing that fermentation with different enzyme concentrations leads to similar outcomes. The separation along PCA1 and PCA2 axes suggests that both fermentation and enzyme treatment significantly impact the overall profile of SBCB. The variable PCA and PCA biplot are presented in Figure 3B,C. Dim1 accounted for 58.3% of the total variance, while Dim2 explained 19.6%. The color gradient, transitioning from blue to red, represents the level of contribution, with red indicating higher contributions. Notably, WF5%, WF3%, F3%, and F5% clustered together, suggesting a strong relationship among these samples (Figure 3B), likely due to the presence of higher concentrations of certain compounds that distinguish them from WEWF and WEF. WEWF and WEF also point in the same direction, indicating closeness, which is due to the presence of ethyl octanoate in these samples only, as reflected in Figure 3C. Although the concentration was relatively low in WEWF. Figure 3C revealed that the compounds in red (acetic acid) and purple (3‐methylbutanoic acid and 3‐methyl butan‐1‐ol) are the major contributors to the variation among SBCB samples, while the compounds in blue have a low contribution. An increase in color intensity tends toward redness, indicating a higher contribution. Acetic acid shows a significant contribution, marked by a large red bubble. The concentration was higher in the fermented samples, particularly the enzyme‐assisted samples. This might explain the overlap observed between these samples. This is expected because *Acetobacter pateurianus* and *Torulaspora delbrueckii* can convert sugars present in these samples to acetic acid.

### 3.5 Mantle Test and Pearson Correlation Heat Map

**FIGURE 4:**
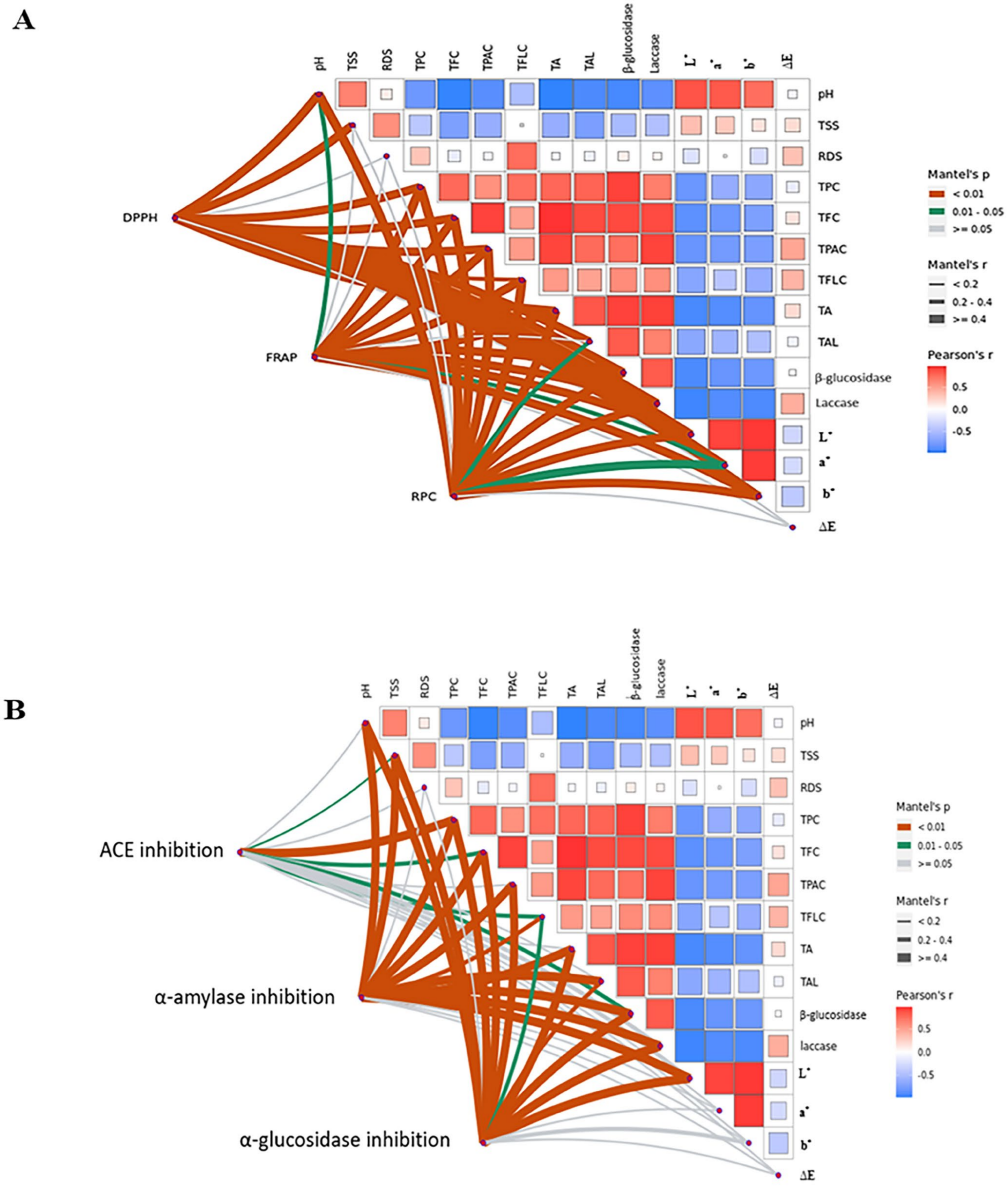
Mantel test showing relationships between antioxidant properties (FRAP, RPC, and DPPH) and phytochemical contents, physicochemical properties, color parameters, and enzyme activity (A), and relationships between antidiabetic (α‐amylase and α‐glucosidase inhibition) and ACE inhibitory properties, and these same factors (B). TSS denotes total soluble solids, RDS denotes reducing sugar, TPC denotes total phenolic content, TFC denotes total flavonoid content, TFLC denotes total flavonols, TPAC denotes total proanthocyanlidin content, TA denotes total acids, TAL denotes total alcohols. Significant correlations based on Mantel's test are marked with outlines: Red for *p* < 0.01, green for 0.01 ≤ *p* < 0.05, and white for *p* ≥ 0.05.

The Mantel test was conducted to thoroughly investigate the relationships between antioxidant properties (FRAP, RPC, and DPPH), and various factors, including phytochemical contents, physicochemical properties, color parameters, and enzyme activity. Furthermore, the correlations between antidiabetic properties (α‐amylase and α‐glucosidase inhibition) and ACE inhibitory activity were investigated. The findings are detailed in Figure 4A,B. FRAP and RPC exhibited strong correlations with all parameters except RDS, TSS, and ∆E, while DPPH correlated strongly with all parameters except RDS and ∆E (Figure 4A). Strong correlations (significant at *p* < 0.01) are indicated by red lines, moderate correlations (0.01 ≤ *p* < 0.05.) by green lines, and nonsignificant correlations (*p* ≥ 0.05) by white lines. The associations between antioxidant properties and phytochemical contents suggest that TPC, TFC, TFLC, and TPAC are major contributors to the antioxidant capacity of SBCB. This is corroborated by previous studies linking antioxidant properties to phytochemical contents (Kwaw et al. 2018; Nwozo et al. 2023). Additionally, Pearson's correlation heatmap reveals a strong negative correlation between pH and both phytochemical contents and enzyme activities. This suggests that lower pH enhances β‐glucosidase and laccase activities, resulting in increased phytochemical contents and improved antioxidant properties. These findings align with those of Kwaw et al. (2017), who attributed increased phytochemical contents to pH reduction and β‐glucosidase activity of the fermenting microorganisms. Regarding ACE inhibition in SBCB, significant associations were found with TPC (*p* < 0.0), and with TSS, TFC, TFLC, and β‐glucosidase (0.01 ≤ *p* < 0.05) (Figure 4B). Al Shukor et al. (2013) have similarly reported ACE inhibitory effects of plant phenolic compounds. Moreover, α‐amylase and α‐glucosidase inhibition showed strong correlations with pH, enzyme activity, phytochemical contents, and L* (Figure 4B). This indicates that TPC, TFC, TFLC, and TPAC significantly contribute to the in vitro antidiabetic potential of SBCB. This conforms with Praparatana et al. (2022), who noted that the antidiabetic properties of *Bauhinia strychnifolia* Craib stem are linked to its phenolic content.

## 4 Conclusion

This research showcases the potential of enzyme‐assisted fermentation as a groundbreaking approach to boost the phytochemical content, bioactivity, and aroma profile of sea buckthorn‐based cereal beverages (SBCB). The combined effect of enzymatic hydrolysis and fermentation substantially increased key phytochemicals and significantly enhanced α‐amylase, α‐glucosidase, and ACE inhibitory activities. Notably, the F3% treatment boosted TPC and TFLC, whereas the F5% treatment elevated TFC and proanthocyanidins. Furthermore, antioxidant properties were considerably improved, and the aroma profile was modified, characterized by increased alcohols and acids and decreased ester production. These results highlight enzyme‐assisted fermentation as a promising approach for creating functional fruit‐based cereal beverages with improved health benefits. The therapeutic potential of SBCB in managing metabolic disorders, including diabetes and hypertension, warrants further exploration to identify active compounds and elucidate the mechanisms underlying their bioactivities. Additionally, optimizing fermentation conditions to balance volatile compound profiles, particularly by promoting ester formation, represents an avenue for improving aroma and overall consumer appeal. This work provides a robust foundation for advancing the application of enzyme‐assisted fermentation in the innovation of plant‐based functional health beverages, with significant implications for both academic research and industrial development. To further substantiate these findings, future studies involving targeted metabolomic analyses and sensory trials are recommended to validate the health benefits and consumer appeal of SBCB.

## Author Contributions

**Afusat Yinka Aregbe:** conceptualization (lead), formal analysis (lead), investigation (lead), methodology (lead), writing – original draft (lead). **Shamas Murtaza:** investigation (supporting). **Ebenezer Ola Falade:** writing – review and editing (supporting). **Md. Hafizur Rahman:** writing – review and editing (supporting). **Yongkun Ma:** conceptualization (supporting), project administration (lead), supervision (lead).

## Conflicts of Interest

The authors declare no conflicts of interest.

## Supporting information


**Figure S1:** Chromatograms of aroma compoundsGC chromatogram of SBCB samples: WEWF (control, untreated), WF3% (unfermented, 3% enzyme‐treated), WF5% (unfermented, 5% enzyme‐treated), WEF (non‐enzyme‐treated, fermented), F3% (fermented, 3% enzyme‐treated), and F5% (fermented, 5% enzyme‐treated). SBCB = sea buckthorn–based cereal beverage.
**Figure S2:** Volatile components of enzyme‐assisted fermented sea buckthorn‐based cereal beverage WEWF (Control) represents untreated SBCB, WF3% represents unfermented, 3% enzyme‐treated SBCB, WF5% represents unfermented, 5% enzyme‐treated SBCB, WEF represents non‐enzyme‐treated, fermented SBCB, F3% represents fermented, 3% enzyme‐treated SBCB, F5% represents fermented, 5% enzyme‐treated SBCB, SBCB represents sea buckthorn–based cereal beverage. Means with different letters are significantly different (*p* < 0.05). TAL means total alcohols, TA means total acid, TES means total esters, TK means total ketones, TAD means total aldehydes, TO means total others.

## Data Availability

Data will be made available upon request.
