# Supplementary material for: Synergistic Effect of Enzyme‐Assisted Fermentation on Phytochemical Content, Volatile Profile, and Antioxidant, Antidiabetic, and ACE Inhibitory Properties of Sea Buckthorn‐Based Cereal Beverages
Source: Food Sci Nutr. 2025 Nov 28;13(12):e71217. doi: 10.1002/fsn3.71217 (PMC12662785; doi:10.1002/fsn3.71217)
Supplement: Supplementary file 1 — Figure S1: Chromatograms of aroma compoundsGC chromatogram of SBCB samples: WEWF (control, untreated), WF3% (unfermented, 3% enzyme‐treated), WF5% (unfermented, 5% enzyme‐treated), WEF (non‐enzyme‐treated, fermented), F3% (fermented, 3% enzyme‐treated), and F5% (fermented, 5% enzyme‐treated). SBCB = sea buckthorn–based cereal beverage. Figure S2: Volatile components of enzyme‐assisted fermented sea buckthorn‐based cereal beverage WEWF (Control) represents untreated SBCB, WF3% represents unfermented, 3% enzyme‐treated SBCB, WF5% represents unfermented, 5% enzyme‐treated SBCB, WEF represents non‐enzyme‐treated, fermented SBCB, F3% represents fermented, 3% enzyme‐treated SBCB, F5% represents fermented, 5% enzyme‐treated SBCB, SBCB represents sea buckthorn–based cereal beverage. Means with different letters are significantly different (p < 0.05). TAL means total alcohols, TA means total acid, TES means total esters, TK means total ketones, TAD means total aldehydes, TO means total others. [file FSN3-13-e71217-s001.docx]

**Fig.S1**


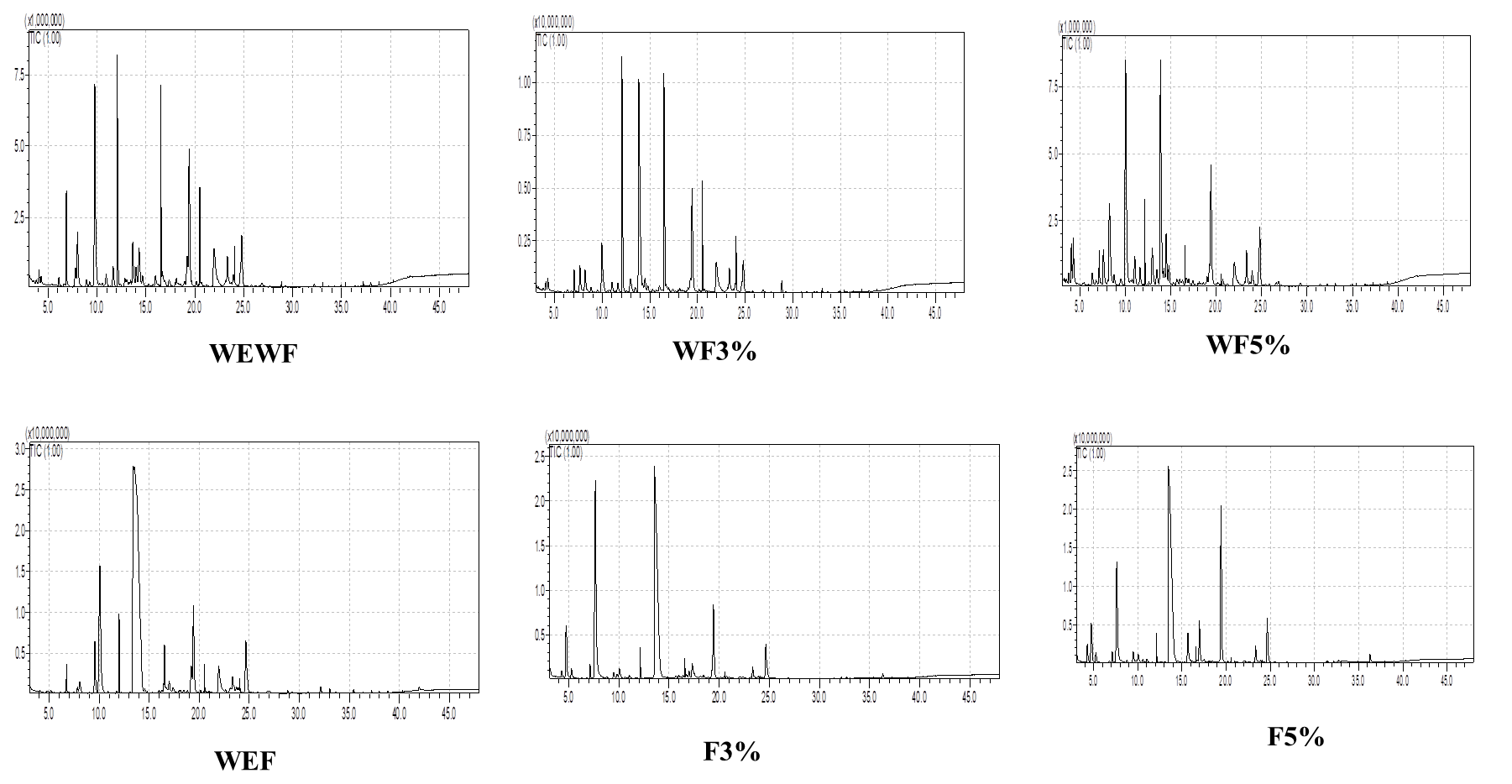


GC chromatogram of SBCB samples: WEWF (control, untreated), WF3% (unfermented, 3% enzyme treated), WF5% (unfermented, 5% enzyme treated), WEF (non-enzyme treated, fermented), F3% (fermented, 3% enzyme treated), and F5% (fermented, 5% enzyme treated). SBCB = sea buckthorn–based cereal beverage.

**Fig.S2**

**
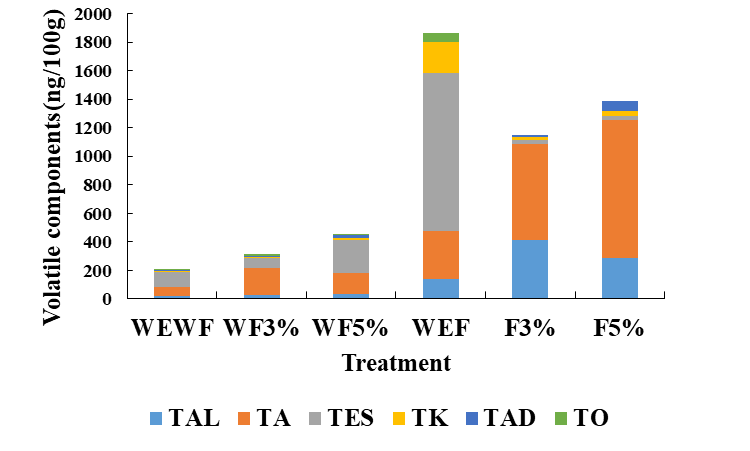
**

WEWF (Control) represents untreated SBCB, WF3% represents unfermented, 3% enzyme treated SBCB, WF5% represents unfermented, 5% enzyme treated SBCB, WEF represents non-enzyme treated , fermented SBCB, F3% represents fermented, 3% enzyme treated SBCB, F5% represents fermented, 5% enzyme treated SBCB, SBCB represents sea buckthorn–based cereal beverage. Means with different letters are significantly different (p ˂ 0.05). TAL means total alcohols, TA means total acid, TES means total esters, TK means total ketones , TAD means total aldehydes, TO means total others
